# Evaluation of the effect of pharmaceutical care during inpatient treatment in a department of neurology: A retrospective study

**DOI:** 10.1097/MD.0000000000030984

**Published:** 2022-10-14

**Authors:** Wen Ji, Ruowei Xiao, Bei Wu, Sheng Han, Jinju Duan, Zhiqiang Meng, Mingxu Yang, Chen Wang

**Affiliations:** a Department of Pharmacy, Second Hospital of Shanxi Medical University, Taiyuan, China; b International Research Center for Medicinal Administration, Peking University, Beijing, China; c Changzhi Medical College, Changzhi, China; d Department of Pharmacy, Shanxi Eye Hospital, Taiyuan, China.

**Keywords:** clinical pharmacist, neurology, pharmaceutical services, role

## Abstract

Common drug-related problems during neurology inpatient treatment can affect expected health results. Some interventions need to be implemented to reduce DRPs. To explore the effect of care from clinical pharmacists during inpatient treatment. Inpatients treated in the department of neurology in the Second Hospital of Shanxi Medical University between January 1 to December 31, 2019, were retrospectively included. Those who received care from the clinical pharmacist service were assigned to the pharma-care group while the other patients were assigned to the control group. From the perspective of drugs, the two groups were compared in terms of types, antimicrobial use, and key monitoring of drug use. From the perspective of patients, the two groups were compared in terms of length of stay, hospital cost, drug cost and proportion. Propensity score matching was used to balance the baseline characteristics. A total of 2684 patients were included 554 in the pharma-care group and 2130 in the control group with a median of 9 days (range, 3–30 days) hospital stay. The groups showed no significant difference in age or gender. Length of stay, the proportion of drug cost, number of adverse events, cost of antibacterial agents, use of a single antibacterial agent, and use of three or more different antibacterial agents were similar between the groups. Medicine expenses cost more in the pharma-care group. The cost and types of intensive monitoring drugs were similar, but Defined Daily Doses were lower in the control group. While clinical pharmacists may play a positive role in the pharmaceutical care of inpatients, in this study the benefits were not obvious. This may be because of the small number of clinical pharmacists in the department of neurology with narrow coverage.

## 1. Introduction

Drugs are the main tools for preventing and treating clinical diseases. At present, there are many drug-related problems (DRPs) in clinical drug therapy.^[1]^ DRPs mainly refer to “pharmacotherapy events that actually or potentially interfere with expected health outcomes”.^[2,3]^ Clinically these events include adverse drug events, adverse drug reactions, and medication errors,^[4]^ which potentially threaten life, affect the quality of life, increase morbidity, and cause readmission^[5,6]^ and medical expenses. The rate of DRPs can be high in some situations. For example, 80% of patients in Turkey have DRPs, and elder age, multi-pharmacotherapy, and complications all increase the incidence of DRPs,^[6,7]^ which can be inevitable even in hospitalized patients.^[8–10]^ Neurology departments are important clinical departments in general hospitals with complex disease types. The spectrum of diseases includes stroke, dementia, and Parkinson’s disease. Patients in the department of neurology are generally older with more basic diseases than other departments and their condition can change rapidly. In a department of neurology in China, inappropriate frequency and medication dose were the most common DRPs (35.8%).^[11]^ This is in contrast to a department of neurology in the UK where compliance with guidelines and contraindications were the most common DRPs (26.3%) that may be related to prescribing errors from junior doctors.^[12]^ Therefore, multiple problems can occur such as the need for many kinds of clinical medications, poor compliance, the increase in hospitalization expense, extension of hospitalization time and adverse reactions.

The majority (50–80%) of DRPs can be prevented.^[13]^ Many countries are trying to reduce DRPs to ensure drug safety and effective disease treatment. Most methods depend on multidisciplinary collaboration. This approach is quite good, but because of the large number of patients in China, multidisciplinary collaboration cannot be achieved in the clinical treatment of every patient. Rather, multidisciplinary collaboration is targeted toward the treatment of critically ill patients and those with rare diseases. In China, the participation of clinical pharmacists in clinical treatment supplements multidisciplinary collaboration and is a new direction for clinical pharmacy.

The approach to including clinical pharmacists in the clinical treatment means that pharmacists actively participate in designing the patients’ pharmacotherapy plans, providing a reference for clinical drug selection according to individualized characteristics, and evaluating the treatment plan for the patients.^[14]^ The pharmacists also collect data on adverse drug reactions; educate patients on medication at their bedside to improve their medication compliance, monitor their medication process until discharge, and assist the patients with the correct administration and dosage of drugs when they are discharged to ensure correct use. Studies have shown that pharmacists’ medication adjustment or discharge medication reports can reduce the number of medication errors after discharge.^[15,16]^ Clinical pharmacists can effectively identify, prevent and solve clinically important DRPs.^[17,18]^ Since clinical pharmacists participate more in clinical treatment, drug-drug interaction decreases and patients’ understanding of drugs is improving, thereby effectively preventing and reducing DRPs.^[14,19,20]^ A review including 14 randomized controlled studies revealed that clinical pharmacist interventions reduced the occurrence of DRPs in elderly patients.^[21]^ Guo et al^[22]^ found that medication reconciliation performed by pharmacist trainees upon admission can reduce unintentional medication discrepancies. Yin et al^[23]^ found that pharmaceutical inpatient care improved adherence in patients with nephrotic syndrome after hospital discharge. Thus, the effectiveness and safety of pharmacotherapy are improved.^[24]^ A pharmacist service was adopted quite early in the department of neurology of our hospital, and the professional skills of the pharmacists have provided clinical assistance and have been praised by doctors.

Most studies to date have focused on the roles of pharmacists, such as the identification and classification of DRPs,^[6,25,26]^ with intervention happening on the recommendations and acceptance of doctors. More direct measurement of the influence of clinical pharmacists is based on observations of the patient’s clinical outcomes. This study aimed to analyze the indicators of drug use and the disease management end-points and to objectively explore the role of pharmacists in the department of neurology.

## 2. Materials and Methods

### 2.1. Study populations

The inpatients treated in the department of neurology of the Second Hospital of Shanxi Medical University from January 1 to December 31, 2019, were retrospectively included.

The inclusion criteria were: age > 18 years, and patients who were conscious and capable of communicating normally without mental disorders.

The exclusion criteria were: patients who died during the study period; pregnant and lactating women; patients with serious diseases in other important organs; patients transferred from other departments in the hospital or to other departments before discharge; patients with the presence of malignancy tumors (main diagnosis as International Classification of Diseases (ICD) with diagnosis code of C00-D48); and incomplete clinical data.

This study was approved by the Ethics Committee of the Second Hospital of Shanxi Medical University. This was a retrospective study, and all the data were retrieved from the medical record system. There was no risk to the subjects, the rights or interests of the subjects were not violated, and the privacy of the subjects was guaranteed. The need for informed consent from the patients was exempted.

### 2.2. Study design

Patients who received pharmacist services during their treatment in the department of neurology were assigned to the pharma-care group, and those who did not receive the pharmacist services were assigned to the control group. Whether the patients could receive pharmacist services depended on their selection of physicians in the outpatient department. Among the five teams of physicians in the department of neurology, only one team included two clinical pharmacists and patients admitted by this team were served by the clinical pharmacists.

Their specific work was as follows: To carefully inquire about the clinical symptoms of patients after drug administration, urge physicians to carry out necessary tests, such as microbial testing, and propose more appropriate administration suggestions for physicians’ treatment decisions^[24]^; To understand the detailed progression of the patients’ diseases, check doctors’ prescriptions, fully communicate with patients, provide patients with bedside medication education, discharge medication education and other pharmaceutical care to ensure the safety of the medication; and To explain the pharmacological effects, indications, contraindications, storage and use of commonly used clinical drugs to the first-line young doctors and nursing staff, to ensure that the dosage and frequency of the drugs met the requirement of treatment.

### 2.3. Clinical data collection

Baseline characteristics of the patients were collected including age, gender, type of health care, smoking status, alcohol consumption, body mass index (BMI), and clinical condition at admission including daily living ability assessed using the Barthel activities of daily living index,^[24]^ consciousness, nutritional condition, critical disease status, number of diseases, and Charlson Comorbidity Index (CCI). The hospitalization information collected included length of stay, total hospitalization cost, self-paying of the total cost, expenses for medicine, the proportion of medical expenses, types of drugs used and intravenous drug use,^[24]^ types of returned drugs, and refund cost. Information on antibacterial agent use collected included cost and types of antibacterial agents, defined daily doses (DDDs) of antibacterial agents, cost of DDDs (DDDc) of unrestricted antibacterial agents, DDDs and DDDc of the special use of antibacterial agents, DDDs and DDDc of limited antibacterial agents, the proportion of antibacterial treatment costs, prophylactic use of antibacterial agents, change in the antibiotic treatment plan, off-label use, bacterial drug sensitivity testing, use of a single antibacterial agent, and use of three or more antibacterial agents. Cost, types, and DDDs of intensive monitoring drugs were also collected.

### 2.4. Statistical methods

Descriptive analyses were performed to compare patients’ baseline characteristics between the pharma-care group and the control group. Categorical variables were expressed as frequency (n) and constituent ratio (%), and a comparison between two groups was performed by χ^2^ test. Almost all of the continuous data showed non-normal distribution and were expressed as median (M) and interquartile range (IQR), and a comparison between two groups was performed by a non-parametric rank-sum test. Propensity score matching (PSM) in a ratio of 1:1 was performed to balance the baseline information between two groups, and a logistic regression was fitted to produce propensity scores by accounting for the covariates, including sex, age, health care type, smoking, drinking, BMI, condition at admission, daily living ability classification, consciousness, critical disease or not, number of diseases, and CCI. A one-to-one nearest-neighbor matching method without replacement and with a 0.01 caliper level was used when PSM was performed. Hospitalization information was further analyzed according to different age groups and in patients with encephalitis, cerebral infarction, and critical or non-critical diseases. *P* < .05 was considered a significant difference. All statistical analyses were done using STATA 14.0 (Stata Corp., Taiyuan, Shanxi, P. R. China.).

## 3. Results

### 3.1. Baseline characteristics

**Figure 1.**
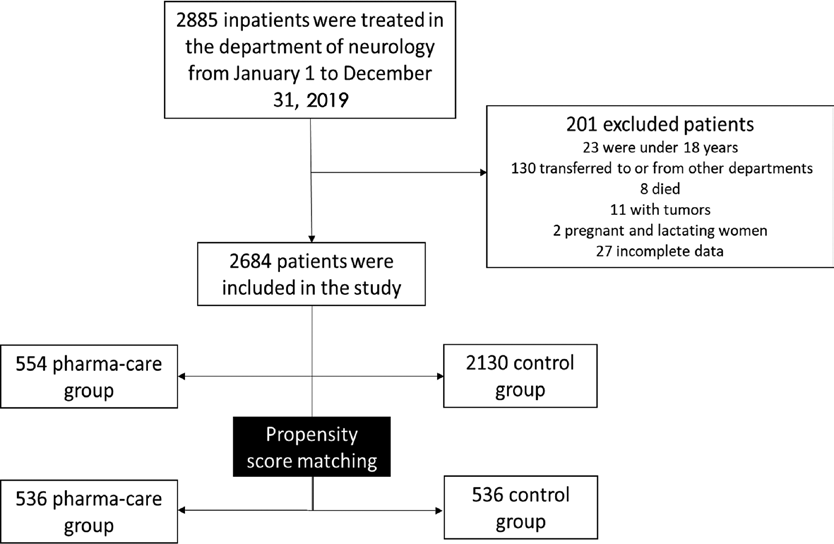
A total of 2885 inpatients were treated in the department of neurology from January 1 to December 31, 2019. However, 201 of them were excluded due to being under the age of 18 (23 inpatients), transferred to or from other departments (130 inpatients), died (8 inpatients), accompanied by another disease like tumors (11 inpatients), pregnant and lactating (2 inpatients), and incomplete data (27 inpatients). Therefore, 2684 inpatients were included in this study in the end. Among these inpatients, 554 of them were divided into the pharma-care group, and the rest of the 2130 patients were divided into the control group. Then, after Propensity Score Matching, 536 patients of the 554 group were assing to the pharma-care group, and 536 patients of the 2130 group were assigned to the control group.

A total of 2885 patients were available during the study period, and finally, 2684 eligible patients were enrolled in the analyses according to the inclusion and exclusion criteria. The patient enrollment flow and study grouping were summarized in Figure 1. The wireframe represents the number and category of patients in this research. The patients are excluded and grouped according to the direction of the arrow. There was no significant difference between the two groups in terms of their sex or age as shown in Table 1. However, there were some differences between the two groups in terms of health care type, body mass index (BMI), patient’s condition at admission, and the patient’s daily living ability (all *P* < .05). Therefore, the two groups were then matched using PSM, leaving 536 patients successfully matched in each group. After PSM, there was no significant difference in covariates between the two groups (Table 1).

**Table 1:** Basic patient information of all patients in the department of neurology.

	Before matching					After matching				
	Pharma-care group (n = 554)		Control group (n = 2130)		*P*	Pharma-care group (n = 536)		Control group (n = 536)		*P*
	N/Median	Proportion/IQR	N/Median	Proportion/IQR		N/Median	Proportion/IQR	N/Median	Proportion/IQR	
Sex										
Male	307	55.4	1238	58.1	.251	300	56.0	308	57.5	.622
Female	247	44.6	892	41.9		236	44.0	228	42.5	
Age (yr)	61.0	19.0	62.5	19.0	.294	62	18.5	61	19.0	.795
≤45	74	13.4	238	11.2						
46–60	187	33.8	707	33.2						
≥61	293	52.9	1185	55.6						
Health care type										
Basic medical insurance	446	80.5	1688	79.3	.136	432	80.6	437	81.5	.982
Reimbursement way	3	0.5	6	0.3		3	0.6	3	0.6	
Self-paying	27	4.9	74	3.5		25	4.7	23	4.3	
Other	78	14.1	362	17.0		76	14.2	73	13.6	
Smoking	155	28.0	632	29.7	.436	151	28.2	158	29.5	.637
Drinking	81	14.6	349	16.4	.313	80	14.9	90	16.8	.403
BMI (kg/m^2^)										
<18.5	20	3.6	94	4.4	<.001	20	3.7	11	2.1	.391
18.5–23.9	211	38.1	852	40.0		202	37.7	205	38.3	
24–27.9	196	35.4	816	38.3		194	36.2	185	34.5	
≥28	56	10.1	255	12.0		54	10.1	66	12.3	
Unknown	71	12.8	113	5.3		66	12.3	69	12.9	
Condition at admission										
Critical	13	2.4	54	2.5	.027	13	2.4	10	1.9	.808
Emergent	15	2.7	116	5.5		15	2.8	16	3.0	
Common	526	95	1960	92		508	94.8	510	95.2	
Daily living ability classification										
No dependence	253	45.7	399	18.7	<.001	242	45.2	243	45.3	.980
Mild dependence	202	36.5	1112	52.2		198	36.9	202	37.7	
Moderate dependence	49	8.8	349	16.4		49	9.1	47	8.8	
Severe dependence	50	9.0	270	12.7		47	8.8	44	8.2	
Consciousness										
Normal	526	95.0	2019	94.8	.954	509	95.0	506	94.4	.637
Disturbance	24	4.3	93	4.4		23	4.3	28	5.2	
Light coma	3	0.5	11	0.5		3	0.6	2	0.4	
Moderate coma	1	0.2	7	0.3		1	0.2	0	0.0	
Nutrition classification										
Risk of malnutrition	0	0.0	4	0.2	.307	0	0.0	2	0.4	.157
Good nutritional status	554	100.0	2126	99.8		536	100.0	534	99.6	
Critical disease	77	13.9	223	10.5	.022	71	13.3	67	12.5	.715
Number of diseases	5	4	5	3	.014	5	4	5	4	.836
CCI_quan	0	2	0	2	<.001	0	2	0	2	.904

Note it was not always possible to accurately measure BMI if the patient was in a wheelchair or laying flat. Therefore, these were recorded as unknown.

BMI = body mass index, CCI = Charlson Comorbidity Index, IQR = interquartile range.

### 3.2. Hospitalization information

No significant difference in length of stay, self-paying of the total cost, types of returned drugs, or refund cost was found between the pharma-care group and the control group (all *P* > .05). However, there was a difference in the hospitalization cost, medicine expenses, the proportion of the expenses being medical expenses, the types of drugs, and the total number of intravenous drugs which were all higher in the pharma-care group and these remained significant in the PSM groups (all *P* < .001) (Table 2).

**Table 2:** Hospitalization information of all patients in the department of neurology.

Item	Before matching					After matching				
	Pharma-care group (n = 554)		Control group (n = 2130)		*P*	Pharma-care group (n = 536)		Control group (n = 536)		*P*
	Median	IQR	Median	IQR		Median	IQR	Median	IQR	
Length of stay (d)	10	4	10	6	.855	10.0	4.5	10.0	6.0	.962
Total hospitalization cost (US dollars)	1668.3	941.7	1516.0	1080.3	<.001	1663.5	947.1	1457.4	1030.0	<.001
Self-paying of the total cost (US dollars)	523.1	750.1	503.2	786.1	.417	517.7	754.9	460.5	756.0	.227
Expenses for medicine (US dollars)	401.5	524.3	324.1	588.6	.001	400.3	523.8	290.9	527.2	<.001
Proportion of medical expenses (%)	23.5	20.5	21.6	22.6	<.001	23.4	20.7	19.7	21.0	<.001
Types of drugs	9.0	5.0	8.0	5.0	<.001	9.0	5.0	8.0	5.0	<.001
Total number of intravenous drugs	3.0	3.0	3.0	2.0	<.001	3.0	3.0	2.0	3.0	<.001
Types of returned drugs	0.0	0.0	0.0	0.0	.538	0.0	0.0	0.0	0.0	.628
Refund cost (US dollars)	0.0	0.0	0.0	0.0	.738	0.0	0.0	0.0	0.0	.676

IQR = interquartile range.

### 3.3. Antibacterial agent use

A comparison of the groups when only considering those who received antibacterial treatment is shown in Table 3. There were 73 in the pharma-care group and 295 in the control group and 68 in each group after PSM. These results showed a significant difference in health care type, BMI, and daily living ability (all *P* < .05) but were not significant after PSM. The most common antibacterial agents used and their DDDs are shown in Supplemental Table 1, http://links.lww.com/MD/H529.

**Table 3:** Basic patient information of inpatients who used antibacterial agents in the department of neurology.

	Before matching					After matching				
	Pharma-care group (n = 73)		Control group (n = 295)		*P*	Pharma-care group (n = 68)		Control group (n = 68)		*P*
	N/Median	Proportion/IQR	N/Median	Proportion/IQR		N/Median	Proportion/IQR	N/Median	Proportion/IQR	
Sex										
Male	41	56.2	167	56.6	.945	38	55.9	43	63.2	.382
Female	32	43.8	128	43.4		30	44.1	25	36.8	
Age (yr)	64	24	66	21	.685	64	23.0	62.5	21.5	.258
Health care type										
Basic medical insurance	58	79.5	229	77.6	.023	54	79.4	49	72.1	.137
Reimbursement way	0	0.0	3	1.0		0	0.0	0	0.0	
Self-paying	7	9.6	8	2.7		7	10.3	4	5.9	
Other	8	11.0	55	18.6		7	10.3	15	22.1	
Smoking	17	23.3	56	76.7	.404	16	23.5	22	32.4	.252
Drinking	7	9.6	44	14.9	.238	7	10.3	10	14.7	.437
BMI (kg/m^2^)										
<18.5	4	5.5	21	7.1	.006	4	5.9	5	7.4	.985
18.5–23.9	28	38.4	121	41.0		25	36.8	27	39.7	
24–27.9	13	17.8	80	27.1		12	17.7	11	16.2	
≥28	5	6.9	33	11.2		5	7.4	4	5.9	
Unknown	23	31.5	40	13.6		22	32.4	21	30.9	
Condition at admission										
Critical	8	11.0	30	10.2	.971	8	11.8	11	16.2	.699
Emergent	6	8.2	23	7.8		5	7.4	6	8.8	
Common	59	80.8	242	82.0		55	80.9	51	75.0	
Daily living ability classification										
No dependence	20	27.4	27	9.2	<.001	16	23.5	16	23.5	.668
Mild dependence	20	27.4	81	27.5		20	29.4	23	33.8	
Moderate dependence	8	11.0	62	21.0		8	11.8	4	5.9	
Severe dependence	25	34.3	125	42.4		24	35.3	25	36.8	
Consciousness										
Normal	59	80.8	231	78.3	.881	54	79.4	53	77.9	.910
Disturbance	10	13.7	50	17.0		10	14.7	9	13.2	
Light coma	3	4.1	9	3.1		3	4.4	4	5.9	
Moderate coma	1	1.4	5	1.7		1	1.5	2	2.9	
Nutrition classification										
Risk of malnutrition	0	0.0	1	0.3	.618	0	0.0	0	0.0	/
Good nutritional status	73	100.0	294	99.7		68	100.0	68	100.0	
Critical disease	31	42.47	123	41.7	.905	31	45.6	33	48.5	.731
Number of diseases	6	5	5	5	.506	6	5	5	3.5	.241
CCI_quan	0	2	0	2	.704	0	2	0	2	.288

BMI = body mass index, CCI = Charlson Comorbidity Index, IQR = interquartile range.

Before PSM there were no significant differences between the two groups in any measured aspect of antibacterial agent use (Table 4, all *P* > .05). However, after PSM there were significant differences in types of antibacterial agents (*P* = .023), proportion of bacterial drug sensitivity (*P* = .030), and single antibacterial agent use rate (*P* = .020) between the two groups.

**Table 4:** Use of antibacterial agents in inpatients in the department of neurology.

Item	Before matching					After matching				
	Pharma-care group (n = 73)		Control group (n = 295)		*P*	Pharma-care group (n = 68)		Control group (n = 68)		*P*
	N/Median	Proportion/IQR	N/Median	Proportion/IQR		N/Median	Proportion/IQR	N/Median	Proportion/IQR	
Cost of antibacterial agents (US dollars)	120.5	212.9	128.5	253.7	.610	136.0	218.3	80.3	203.5	.166
Types of antibacterial agents	1.0	1.0	1.0	1.0	.937	1.0	1.0	1.0	0.0	.023
Antibacterial agents DDDs	7.0	7.6	6.3	8.5	.388	7.0	8.0	5.5	8.6	.108
Unrestricted antibacterial agents DDDs	0.0	0.1	0.0	0.2	.968	0.0	0.0	0.0	0.4	.472
Unrestricted antibacterial agents DDDc	0.0	1.7	0.0	1.1	.972	0.0	0.0	0.0	1.7	.561
Special use of antibacterial agents DDDs	0.0	0.0	0.0	0.0	.403	0.0	0.0	0.0	0.0	.739
Special use of antibacterial agents DDDc	0.0	0.0	0.0	0.0	.374	0.0	0.0	0.0	0.0	.747
Limited antibacterial agents DDDs	5.7	10.0	4.9	7.6	.305	5.8	9.6	3.9	6.9	.103
Limited antibacterial agents DDDc	104.0	124.1	111.1	187.4	.058	104.0	136.0	104.0	203.2	.972
Antibacterial agent use for treatment	44	60.3	186	63.1	.661	42	61.8	38	55.9	.486
Antibacterial agent use for prevention	29	39.7	109	37.0	.661	26	38.2	30	44.1	.486
Treatment plan changed	22	30.1	77	26.1	.486	21	30.9	12	17.6	.070
Off-label use	3	4.1	8	2.7	.530	3	4.4	3	4.4	1.000
Bacterial drug sensitivity testing	46	63.0	167	56.6	.321	45	66.2	33	48.5	.030
Use of single antibacterial agent	47	64.4	189	64.1	.960	43	63.2	55	80.9	.020
Use of three or more antibacterial agents	3	4.1	11	3.7	.879	3	4.4	2	2.9	.640

DDDc = cost of DDDs, DDDs = defined daily doses, IQR = interquartile range.

### 3.4. Intensive monitoring of drug use

The cost, types, and DDDs of intensive monitoring drugs used by the inpatients are shown in Table 5. Before PSM they were all significantly lower in the control group; however, after PSM only the DDDs remained significantly different between the groups (*P* < .001). The most common monitored drugs used and their DDDs are shown in the Supplemental Table 1, http://links.lww.com/MD/H529.

**Table 5:** Use of monitored drugs for inpatients in the department of neurology.

Item	Before matching					After matching				
	Pharma-care group (n = 512)		Control group (n = 1783)		*P*	Pharma-care group (n = 496)		Control group (n = 496)		*P*
	Median	IQR	Median	IQR		Median	IQR	Median	IQR	
Cost of intensive monitoring drugs (US dollars)	204.3	228.1	173.2	249.7	<.001	203.7	221.7	196.3	251.7	.142
Types of intensive monitoring drugs	2.0	1.0	2.0	1.0	.001	2.0	1.0	2.0	1.0	.066
Intensive monitoring drugs DDDs	15.4	15.6	10.5	13.3	<.001	15.3	15.8	11.7	15.8	<.001

DDDs = defined daily doses, IQR = interquartile range.

### 3.5. Differences among age groups

When the data were analyzed according to different age groups, there were significant differences in the proportions of medical expenses (*P* = .005), types of drugs used (*P* < .001), and the types of intravenous drugs (*P* < .001) used by inpatients who were aged >60 years, namely a higher proportion and more types of drugs were used in the pharma-care group. There were also significant differences in the types of drugs (*P* = .005) used and the types of intravenous drugs (*P* = .036) used by inpatients aged 45 to 60 years, namely, there were more types of medications used in the pharma-care group (Table 6).

**Table 6:** Hospitalization information according to different patient age groups.

Item	≤45 years old					46–60 years old					>60 years old				
	Pharma-care group (n = 74)		Control group (n = 238)		*P*	Pharma-care group (n = 187)		Control group (n = 707)		*P*	Pharma-care group (n = 293)		Control group (n = 1185)		*P*
	Median	IQR	Meduan	IQR		Median	IQR	Median	IQR		Median	IQR	Median	IQR	
Length of stay (d)	8.5	4.0	9.0	6.0	.459	10.0	5.0	10.0	6.0	.246	10.0	4.0	10.0	6.0	.245
Proportion of medical expenses (%)	19.2	17.7	15.6	23.3	.372	21.9	18.3	21.4	22.4	.464	25.8	21.5	23.1	22.9	.005
Types of medicine	7.0	6.0	7.0	5.0	.127	9.0	5.0	8.0	6.0	.005	10.0	5.0	9.0	5.0	<.001
Types of intravenous drugs	3.0	3.0	3.0	3.0	.148	3.0	2.0	3.0	2.0	.036	3.0	3.0	3.0	2.0	<.001

IQR = interquartile range.

### 3.6. Hospitalization information of patients with encephalitis, cerebral infarction, and critical or non-critical diseases

There was no significant difference between the pharma-care and control groups in patients with encephalitis in the length of hospital stay (*P* = .545). However, the proportion of medical expenses (*P* = .011), type of medicine (*P* < .001), and types of intravenous drugs (*P* < .001) were different. There were also significant differences in the types of drugs and intravenous drugs used between the two groups in inpatients with cerebral infarction (both *P* < .001). More types of drugs and intravenous drugs were used in the pharma-care group than in the control group for patients with both diseases (Table 7).

**Table 7:** Hospitalization information of patients with specific diseases.

Item	Encephalitis					Cerebral infarction				
	Pharma-care group(n = 21)		Control group (n = 68)		*P*	Pharma-care group (n = 187)		Control group (n = 865)		*P*
	Median	IQR	Median	IQR		Median	IQR	Median	IQR	
Length of stay (d)	12	6.0	12.0	6.5	.545	11.0	4.0	11.0	4.0	.773
Proportion of medical expense (%)	25.0	12.3	18.8	15.5	.011	35.3	22.2	35.7	23.8	.977
Types of medicine	14.0	7.0	9.5	7.0	<.001	11.0	5.0	9.0	5.0	<.001
Types of intravenous drugs	8.0	4.0	5.0	3.0	<.001	4.0	2.0	3.0	3.0	<.001

IQR = interquartile range.

When the patients were analyzed according to whether they had critical or non-critical diseases, the results showed that length of stay, the proportion of medical expenses, types of medication and types of intravenous drugs were similar between the pharma-care group and control group in patients with critical diseases (all *P* > .05). In patients with non-critical diseases, more types of medication and types of intravenous drugs were used in the pharma-care group compared to the control group (both *P* < .001) (Table 8).

**Table 8:** Hospitalization information of patients with critical diseases in the department of neurology.

Item	Non-critical diseases					Critical diseases				
	Pharma-care group (n = 477)		Control group (n = 1907)		*P*	Pharma-care group (n = 77)		Control group (n = 223)		*P*
	Median	IQR	Median	IQR		Median	IQR	Median	IQR	
Length of stay (d)	9	4.0	10.0	5.0	.514	13.0	4.0	13.0	6.0	.219
Proportion of medical expenses (%)	22.0	19.0	20.8	22.1	.059	34.1	23.5	28.9	23.5	.100
Types of medicine	9.0	5.0	8.0	5.0	<.001	14.0	9.0	12.0	8.0	.084
Types of intravenous drugs	3.0	2.0	2.0	2.0	<.001	7.0	6.0	6.0	4.0	.902

IQR = interquartile range.

## 4. Discussion

This study aimed to investigate the effect of care from clinical pharmacists on patients in the department of neurology in our hospital. 554 patients in the pharma-care group received pharmacist support in the department and 2130 patients in the control group did not receive dedicated support from a pharmacist. Length of stay, the proportion of self-paying of the total hospitalization cost, cost of antibacterial agents, DDDs of antibacterial agents, DDDs and DDDc of unrestricted antibacterial agents, special use of antibacterial agents, and limited antibacterial agents were similar between the groups. There was also no statistical difference between the groups in terms of types and DDDs of intensive monitoring drugs, the proportion of medical expenses in patients >60 years old and in patients with cerebral infarction, and types of medicine and intravenous drugs in patients <60 years old and in patients with critical diseases. However, medicine expenses were higher in the pharma-care group and the proportion of medical expenses, types of drugs, the total number of intravenous drugs, and DDDs of intensive monitoring drugs. These results suggest that no clear benefit, such as shortening hospital stay or decreasing the expenses for medicine, was found in the patients who received pharmacist care in our hospital.

Previous studies investigating the role of pharmacists in various hospital departments and adult and pediatric patients have shown that their care can decrease the rate of drug-related problems and improve patient outcomes.^[25,26]^ However, other studies have found that while the incidence of drug-drug interactions or DRPs may decrease there was no obvious difference in clinical outcomes.^[27,28]^ Optimizing pharmacotherapy in the form of drug selection and avoiding major drug-drug interactions may inhibit disease complications and shorten hospital stays.^[27,28]^ The inconsistency in the results suggests that there might be many factors that influence the impact of pharmacists in clinical practice. In the present study, most objective indicators were similar between the two groups, probably because the number of patients who received pharmacist intervention was too small to show subtle differences in these outcomes. A more complete pharmacist service in the neurology department might provide better care.

The lack of obvious improvement in objective indicators of patients with pharmacist care in this study may also result from certain difficulties in the provision of this service. For example, the time for pharmacists to communicate with patients and provide valid service was limited. So that even when the service was provided, the impact was not significant. There could be various reasons. Firstly, even though the clinical pharmaceutical care system in China is undergoing rapid development and change, there remain too few clinical pharmacists, so the number of patients who can receive pharmacist care is limited. In general, clinical pharmacists can only serve one-third of the patients in a neurology department. Secondly, there are no standard service responsibilities currently in China, supervision or assessment for the work of clinical pharmacists. The working hours of clinical pharmacists cannot be guaranteed and direct clinical pharmacy work can account for less than 60% of a pharmacist’s working hours.^[29]^ Thirdly, while pharmacists can help educate patients about appropriate medication use and improve their compliance,^[30–32]^ the chance to provide this assistance may be limited during a short hospital stay with little oral medication.

Pharmacists have limited clinical experience. The undergraduate education of pharmacists mainly focuses on chemistry and pharmacy rather than the application of drugs. Pharmacists have to receive continuing education when they are on the job, but it usually aims at accomplishing the task and is often superficial, providing little improvement in capacity for pharmaceutical service and clinical treatment. In addition, there is no systematic training system which focuses on the practice of clinical medicine. Therefore, a different approach to training may be needed.^[33]^

Physicians and patients generally have low recognition of pharmacists, which has been reported by previous studies in different countries. Discussions with physicians can be difficult when many pharmacists have little contact with clinical practice and lack clinical experience, while most doctors use drugs based on their previous experience. So, discourse and suggestions between pharmacists and physicians are also reduced. Neurology inpatients of a large Swiss university hospital^[34]^ reported that 62% of the recommendations from the pharmacists were accepted by the neurologists. In 2008, Ming Hu, Lingli Zhang, et al conducted a sampling survey in 310 hospitals in China,^[35]^ which reported that doctors believed “pharmacists have no ability and level to participate in clinical practice”, and “clinical pharmacists have no role to play”, “developing clinical pharmacy will affect their earnings”, “clinical pharmacists have no qualification to participate in clinical practice”. Compared with 10 years ago, doctors in tertiary hospitals have improved their attitude towards clinical pharmacists and clinical pharmaceutical care,^[36]^ but they still do not completely trust the advice from pharmacists. At the same time, the medical culture is deeply rooted, and patients have absolute trust in doctors, while the social recognition of pharmacists’ value in clinical treatment is much lower. Pharmacists have less time in the clinic and less contact with patients, and patients have much less trust in pharmacists than doctors.^[29]^ Therefore, both physicians and patients should take the initiative to adopt the suggestions or medication plans proposed by pharmacists, which may improve the role of pharmaceutical care in clinical treatment. Thus, the hospital should develop a team of hospital pharmacists, strengthen their training in pharmaceutical care, and provide more patients with professional medication guidance, to ensure the safety and standardization of medication, fully playing the role of pharmacotherapy, reduce the cost and economic burden of patients.

The finding of this study that medication costs were higher in the patients who received pharmacist support is quite surprising. Previous studies have suggested that pharmacist intervention could sometimes provide cost savings in medication costs.^[37–39]^ A study from Canada in an emergency department showed that while pharmacist intervention caused an increase in direct medication, this led to an overall decrease in costs.^[40]^ It is not clear why pharmacist intervention resulted in higher medical costs in this study, but it could be that more effective drugs are more expensive. If this was the case further studies with more detailed indicators might expect to find differences in patient outcomes. This was not seen in terms of hospital stay in this study possible for the reasons already discussed.

The results of this study do suggest that pharmacist support may have assisted in certain aspects of patient care. Older age is a high-risk factor for DRPs because older patients have multiple complications and types of medication. DRPs are particularly associated with certain medications such as analgesics, antiplatelet agents, anticonvulsants, and lipid-lowering agents.^[34]^ A study into neurological care in China showed that drugs for hyperhomocysteinemia, stress ulcer prevention, poor circulation, lowering blood glucose levels, infection, and antiplatelets were highly likely to be related to medication errors.^[11]^ The results indicated good prescription practice and awareness of guideline recommendations by neurologists. Clinical pharmacists can also help identify DRPs in prescription drugs for non-specialist diseases. For example, the rational antibiotic prescription is highly important to prolong the life expectancy of patients with infection in neurology wards, providing evidence of the positive influence of specialized services in neurology with clinical pharmacist services.^[24]^

Overall, if a pharmacist system is to become more effective during clinical treatment, we suggest three main points need to be addressed. Establish a sound pharmacist system and give pharmacists more right to share their opinion in clinical treatment; Increase clinical practice during pharmacist education and improve the efficiency of continuing education to improve the basic skills of pharmacists in clinical treatment; and Ensure pharmacists have enough time to provide the service, for example, let pharmacists be resident in a department, instead of only providing service for patients when needed.

Limitations of the study: This was a single-center study, and the patients were not randomly allocated into the two groups, which may lead to some bias in the results. Because of the retrospective study nature, some important details were missing in the records of some patients and could not be analyzed, including adverse drug reactions. The standard practice involving pharmacist reviewers, doctor’s advice reviews, antibacterial training, and pharmacists’ participation in various consultations, such as anti-infection, critical illness consultation and other pharmaceutical work carried out in the whole hospital was provided for both groups, which narrowed down the difference between the two groups and might be one of the reasons for the negative results. The long-term effect of clinical pharmacist intervention on patient outcomes was not assessed. Future studies should focus on assessing the outcomes of patients undergoing long-term intervention by clinical pharmacists in randomized controlled trials.

## 5. Conclusion

Clinical pharmacists should play a positive role in anti-infection, monitoring drug use and pharmacotherapy of individual patients. However, the results of this study did not show any difference in hospitalization information including length of stay and expenses for medicine between those who received pharmacist care and those who did not. The possible reason might be the small number of clinical pharmacists with narrow coverage, thus even when the support was provided the impact was limited. Therefore, a sound pharmacist system should be established to cultivate more pharmacists who can provide effective services for clinical treatment.

## Acknowledgments

We thank the medical records office and information department of Shanxi Medical University who assisted in the collection of medical records, and assistance from Kai Wu who assisted with data collection and recording DRPs in this study.

## Author contributions

**Conceptualization:** Wen Ji, Sheng Han, Chen Wang.

**Data curation:** Wen Ji, Ruowei Xiao, Bei Wu, Zhiqiang Meng.

**Formal analysis:** Ruowei Xiao.

**Investigation:** Wen Ji, Zhiqiang Meng, Mingxu Yang, Chen Wang.

**Methodology:** Wen Ji, Ruowei Xiao, Bei Wu, Sheng Han, Chen Wang.

**Project administration:** Bei Wu, Chen Wang.

**Resources:** Bei Wu.

**Software:** Ruowei Xiao.

**Supervision:** Wen Ji, Sheng Han, Jinju Duan, Chen Wang.

**Validation:** Ruowei Xiao, Chen Wang.

**Visualization:** Ruowei Xiao.

**Writing – original draft:** Wen Ji, Bei Wu, Mingxu Yang.

**Writing – review & editing:** Bei Wu, Sheng Han, Jinju Duan, Chen Wang.

## Supplementary Material


