# Supplementary material for: Evaluation of the effect of pharmaceutical care during inpatient treatment in a department of neurology: A retrospective study
Source: Medicine (Baltimore). 2022 Oct 14;101(41):e30984. doi: 10.1097/MD.0000000000030984 (PMC9575809; doi:10.1097/MD.0000000000030984)
Supplement: Supplementary file 1 [file medi-101-e30984-s001.pdf]

Supplementary Table 1. DDDs of antibacterial agents and monitored drugs (the first 10 types)

Antibacterial gents	DDDs	Monitored drugs	DDDs
Ceftriaxone	954.5	Edaravone	9798.2
Cefoperazone sulbactam	512.3	Vinpocetine	7306.0
Meropenem	365.3	Alprostadil	6836.0
Levofloxacin	304.0	Ginkgo biloba extract	5889.2
Piperacillin shubatan	196.8	Oxiracetam	1516.4
Moxifloxacin	194.0	Omeprazole	1456.0
Azithromycin	129.0	Lansoprazole	224.0
Penicillin sodium	120.0	Esomeprazole	37.3
Amoxicillin	115.0	Monosialotetera- hexosyl ganglioside	21.3
Cefuroxime	69.0	Cinepazide	1.0

Abbreviation: defined daily doses (DDDs).
